# The role of epigenetic modifications in sensory hair cell development, survival, and regulation

**DOI:** 10.3389/fncel.2023.1210279

**Published:** 2023-06-14

**Authors:** Ying Xiao, Dan Li

**Affiliations:** Department of Otorhinolaryngology, Union Hospital, Tongji Medical College, Huazhong University of Science and Technology, Wuhan, China

**Keywords:** epigenetic regulation, inner ear, sensory hair cell, hair cell protection, hair cell regeneration

## Abstract

The cochlea is the sensory organ in the periphery, and hair cells are its main sensory cells. The development and survival of hair cells are highly controlled processes. When cells face intracellular and environmental stimuli, epigenetic regulation controls the structure and function of the genome in response to different cell fates. During sensory hair cell development, different histone modifications can induce normal numbers of functional hair cells to generate. When individuals are exposed to environmental-related hair cell damage, epigenetic modification also plays a significant role in the regulation of hair cell fate. Since mammalian hair cells cannot regenerate, their loss can cause permanent sensorineural hearing loss. Many breakthroughs have been achieved in recent years in understanding the signaling pathways that determine hair cell regeneration, and it is fascinating to note that epigenetic regulation plays a significant role in hair cell regeneration. In this review, we discuss the role of epigenetics in inner ear cell development, survival and regeneration and the significant impact on hearing protection.

## Introduction

Epigenetics refers to heritable changes in gene expression without changing the DNA sequence. It can regulate the function of genes through chemical modification of DNA and histones, non-coding RNA expression and chromatin remodeling (Henikoff and Matzke, 1997). Epigenetic regulation can maintain the stability of genome structure, normal embryo development and cell differentiation. Abnormal DNA methylation and histone modification can lead to a variety of diseases, including type 2 diabetes, metabolic syndrome and cancer (Egger et al., 2004). Epigenetic regulation can affect the differentiation, proliferation and survival of cochlear hair cells (HCs). Hearing loss caused by HC death is permanent because the HCs in the mammalian cochlea cannot regenerate. When HCs suffer from different injury factors, a variety of intracellular responses, such as apoptosis and inflammation pathways, are initiated. Epigenetic modification plays an important role in acquired deafness, with some modifications protecting HCs and others inducing HC damage. Recently, researchers found that supporting cells (SCs) can transfer to HC progenitors after signaling pathway regulation. Epigenetic regulation is essential for the regeneration of some organs and participates in inner hair cell regeneration pathway regulation. Here, we present a brief review of the role of epigenetic regulation in HC survival and regeneration.

## Epigenetic regulation in the cell cycle

Deoxyribonucleic acid methylation and histone modification are the main regulatory forms of epigenetics. DNA methylation is catalyzed by DNA methyltransferase, which can methylate the cytosine in CpG dinucleotides to 5-methylated cytosine. Generally, CpG islands exist in an active form that is unmethylated, and DNA methylation can cause gene expression silencing (Corso-Díaz et al., 2018). In other words, cytosine methylation is usually correlated with inverse gene expression. DNA methylation is essential for maintaining the stability of cell function, such as imprinting, stabilization of genomic structure, inactivation of X chromosomes in female individuals, normal development of embryos and cell differentiation (Sasaki and Matsui, 2008; Bogdanović and Lister, 2017). During the embryo and mammalian somatic cells development, DNA methylation is removed quite early stage in development. In the pluripotent cell differentiation progress, DNA methylation causes pluripotency-associated and developmental genes suppression, and pluripotency-related genes start to be expressed (Reik, 2007). Histone modifications include acetylation, methylation, phosphorylation, ubiquitination, and so on. Acetylation and methylation are the most studied forms of histone modification. Histone modification can cause nucleosome structure changes, which can cause chromatin remodeling and affect the binding of transcription factors with DNA. Generally, histone acetylation marks the activation of chromosome transcription, and low acetylation or deacetylation of histones indicates that euchromatin regions are in a non-transcriptionally active state. The ubiquitin-proteasome system (UPS) could control CDK-cyclin complexes levels during the cell cycle by the ubiquitin ligases. Ubiquitin ligases could affect cell cycle by participate the degradation of several cell cycle regulators such as cycle D, CKIs, and p21. Cell cycle activation and progression depend largely on UPS, and disruption of this pathway can cause abnormal cell cycle and death in cells (Requejo-Aguilar, 2023).

## Epigenetic regulation of cell regeneration

Induced pluripotent stem cells (iPSCs) are primitive undifferentiated cells with multidirectional differentiation potential and self-replication ability. Sequencing analysis showed that the expression of four transcription factors was changed in iPSC cells: octamer-binding transcription Factor 4 (Oct4), sex-determining region Y-Box 2 (Sox2), Krüppel Like Factor 4 (KLF4), and the oncogene c-MYC (Takahashi and Yamanaka, 2006). The increased expression of these four transcription factors induces the upregulation of gene expression related to DNA replication and cell proliferation. The regulatory elements of the *Oct4* gene were well regulated by DNA methylation. In embryonic stem cells, *Oct4* gene regulatory elements are unmethylated and play a key role in stem cell development. In mouse embryonic fibroblasts, iPSCs were observed with robust reactivation of pluripotency when two methyltransferase genes, *Dnmt3a* and *Dnmt3b*, were conditionally knocked out (Pawlak and Jaenisch, 2011). In mature DRG neurons, inhibiting the enzymatic activity of HDACs could induce the expression of multiple regeneration-associated genes after spinal cord injury and promote axon regeneration (Finelli et al., 2013). In human endometrial microvascular endometrial cells, KLF4 could regulates angiogenesis via epigenetic regulation. The succinyltransferase GCN5 could interacts with KLF4 and binding to the promoter region of vascular endothelial growth factor A to succinylate H3K79 which could initiate gene transcription (Cao et al., 2022). Furthermore, in breast tumor, PRMT5 could arginine methylation of KLF4 and elevating its protein levels, which could suppress tumor initiation and progression (Hu et al., 2015). Forkhead factors are well-known epigenetic regulators that can interacting with a chromatin remodeler or heterochromatin to relaxing the chromatin landscape. In cardiovascular development, FOXK1 could transcriptionally and epigenetically repressing Wnt signaling in cardiac progenitor cells, especially inhibit WNT6 expression (Sierra-Pagan et al., 2023). The deletion of Kdm6a, an H3K27me3 demethylase, in chondrocytes inhibits the transcription of Wnt10a and Fzd10. Wnt10a signaling suppression exhibiting chondroprotective by preserves chondrocytic activity and reducing osteoarthritic degeneration (Lian et al., 2023). The olfactory placode GnRH neurone development accompanied with timely epigenetic modifications to the Fgf8 locus which were crucial for down-stream transcriptional activation and repression. In the early embryo, TET1 will binding to Fgf8 promoter CpG islands and catalyzes DNA demethylation together with H3K4me3 chromatin unwinding to promote Fgf8 transcription. With the olfactory placode developing, polycomb repressive complex-2 recruitment and H3K27me3 induced chromatin condenses and repression of Fgf8 transcription (Linscott and Chung, 2020).

## The role of epigenetics in the regulation of hair cell development and survival

The development of the inner ear is a dynamic and complex process that involves the differentiation of the ectodermal epithelium, the formation and remodeling of the auditory sac and the differentiation of the sensory and non-sensory epithelium. Inner ear induction and development require the precise regulation of many genes and transcription factors. Many diseases with hereditary hearing loss are caused by gene mutations whose proteins control the chromatin state, such as those involved in DNA methylation, histone modification, or chromatin remodeling (Layman and Zuo, 2015). Similar to Wolf-Hirschhorn syndrome, haploinsufficiency in *Whsc1* causes HC shape, arrangement, morphological specialization and innervation to be disrupted. This suggests that dysfunction of WHSC1, a histone methyltransferase that catalyzes H3K36 methylation, can induce sensorineural hearing loss in syndrome (Doetzlhofer and Avraham, 2017).

Cochlear development is a well-established link between Wnt signaling pathways, including otic induction, planar orientation of the stereocilia and chondrogenesis of the otic capsule (Munnamalai and Fekete, 2013). In the otocyst development, Wnt/β-catenin and FGF signaling coordinated restricting the size of otic placode. Wnt activation causes the otic cells expand and reduce the epibranchial domain (Jayasena et al., 2008). *Atoh1* is a Wnt target gene which control the differentiation of sensory hair cells. ATOH1 is continue expressed in HCs during develop and down-regulated in SCs (Zheng and Gao, 2000). Activating Wnt activity could increase ATOH1 expression and produced extra HCs formation. On the other hand, suppressing Wnt activity reduced ATOH1 expression and restricted the growth of HCs. Hence, the Wnt pathway acts upstream of ATOH1 in cochlea developing and encourages the HCs development by increasing ATOH1 expression (Żak et al., 2015).

The development of spiral ganglion neurons depends upon *Eya1*, Sox2, and *Neurog1* (Elliott et al., 2021). *Neurog1* is an upregulator of *Neurod1* and *Atoh1*, and the total length of the cochlea was reduced in *Neurog1*-null mice (Matei et al., 2005). Sensory neuron formation was completely abrogated in *Neurog1*-null mice (Ma et al., 1998). Loss of *Neurod1* caused prolonged *Atoh1* expression and switched the differentiation fate of sensory neurons (Matei et al., 2005). At the onset of HC differentiation, *Atoh1* expression was upregulated, inhibitory H3K27me3 was reduced, and H3K9ac was increased. During HC maturation, the downregulation of *Atoh1* mRNA was accompanied by a decrease in H3K9Ac and the acquisition of H3K9me3 (Samarajeewa et al., 2019). Inhibition of acetylation downregulates the expression of *Atoh1* mRNA in nascent HCs and blocks HC differentiation in embryonic organ of Corti development *ex vivo* (Stojanova et al., 2015).

The fibroblast growth factor (FGF) signaling pathway plays a role in multiple stages of inner ear development, such as the formation of auditory placodes and otocysts and the proliferation and differentiation of sensory epithelial cells. Inhibition of *FGF20* expression in mouse auditory epithelial cells resulted in a decrease in the number of HCs and SCs, and simultaneous loss of *FGF3* and *FGF8* resulted in abnormal development of otocysts (Hayashi et al., 2008; Jacques et al., 2012). As a necessary factor for the processing of double-stranded RNA into small-interfering RNAs, the function of *Dicer1* is crucial for the processing of pre-miRNA into functional mature miRNAs (Bernstein et al., 2003). In *Dicer1 otic* mutants, *Fgf10* expression was severely downregulated and resulted in cochlear outgrowth defects (Bernstein et al., 2003; Pauley et al., 2003).

Histone modification plays a critical role in neurodegeneration and presbycusis. In the cochlea of aging mice, acetylated histone H3 was changed to demethylated histone H3 from youth to aged (Watanabe and Bloch, 2013). In D-galactose-induced aging mice, the expression of connexin 26, a major protein subunit that forms gap junctions in the cochlea, decreases during the aging process. The bisulfite sequencing PCR results showed that the CpG sites of fragment 1 in the connexin 26 gene promoter region were hypermethylated after D-gal treatment, which may induce connexin 26 inactivation and aging-induced hearing loss development (Wu et al., 2014). A cross-sectional study indicated that cardiovascular measurements established that epigenetic clocks were associated with hearing loss. This may be due to subclinical cardiovascular disease changes affecting the cochlear microvascular environment and hearing ability (Kuo et al., 2021).

Epigenetic regulation also plays an important role during acquired hearing loss. N6-methyladenosine (m6A) is a common internal modification that regulates the transcriptome of eukaryotic cells by influencing mRNA metabolism. In cisplatin (CDDP)-induced HC damage, the m6A-binding protein YTHDF1 was increased after CDDP treatment, and its activation could promote cell survival by activating the autophagy pathway. Knockdown of YTHDF1 expression attenuated ATG14 expression, causing autophagy impairment and cell apoptosis. However, overexpression of YTHDF1 alleviated CDDP-induced HC damage. The data suggested that m6A methylation could participate in autophagy regulation and affect HC survival facing CDDP injury (Huang et al., 2022). During gentamycin- and kanamycin-induced HC damage, the drug will reduce histone acetylation and increase deacetylases in OHCs, and histone deacetylase (HDAC) inhibitors could protect against ototoxic drug-induced HC loss *in vitro.* However, these inhibitors failed to exhibit HC protective effects *in vivo*. The results indicate that HDAC inhibitors could attenuate aminoglycoside-induced ototoxicity in an acute model instead of chronic cochlear damage (Chen et al., 2009; Yang et al., 2017). Another group also reported that the HDAC inhibitor SAHA could almost completely protect HCs against acute kanamycin insult. SAHA protection was associated with RelA acetylation (K310) and subsequent Nf-κB pro-survival pathway activation, which could activate the following pro-survival pathway *Cflar/cFLIPL* and *Bcl2l1/Bcl-xL* (Layman et al., 2015). After exposure to traumatic noise, the H3K9ac level decreased in cochlear tissues. Treatment with the pan-HDAC inhibitor SAHA could partly reduce HC loss and hearing loss caused by noise trauma (Chen et al., 2016). Furthermore, the key histone lysine methyltransferase G9a, which is responsible for H3K9me2, is increased in the thymus, spiral ganglion neurons and lateral wall after noise exposure. Inhibition of G9a by BIX01294 or inner ear siRNA injection could reduce the loss of HCs and ribbon synapses to rescue hearing after noise exposure. G9a inhibition could prevent noise-induced KCNQ4 diminishment (Xiong et al., 2019). In the ototoxic drug damage model, global H3K9me2 levels increased significantly during HC damage. After pretreatment with BIX01294 to block the increase in H3K9me2, the Corti organ explant cultures exhibited resistance to cisplatin and aminoglycoside-induced HC damage. The suppression of the activation of the mitochondrial apoptosis pathway may be responsible for the protective effect of BIX01294 on HCs (Yu et al., 2013). The TMRM staining were used to detect mitochondrial function and it shows that BIX01294 is able to prevent the mitochondrial membrane potential disruption caused by neomycin. However, further research is required to fully explore the effect of G9a/GLP inhibition and consequent H3K9me2 reduction on mitochondrial function.

## Regeneration pathway of cochlear hair cells

Sensorineural hearing loss is commonly caused by environmental damage and genetic defects in inner ear sensory epithelia or HCs. In mammals, cochlear HCs cannot regenerate after damage, so HC loss will permanently cause hearing loss. However, HCs can be regenerated in fish and birds. A chicken otocyst culture system was used to study inner ear cell development, and the generation of HCs and SCs was noticeably inhibited when BMP signaling was inhibited. When exogenous BMP4 was administered in culture, there was a significant increase in the number of HCs compared with the untreated group, but it induced prospective inner ear ganglion cell apoptosis. This suggests that BMP4 may increase the number of HCs by encouraging the differentiation of sensory epithelium progenitor cells. These results show that BMP4 could influence several cell populations of the developing inner ear in regards to cell proliferation and death (Li et al., 2005). In zebrafish sensory HCs, scRNA-seq were performed to track the HCs regeneration progress after injury. Researchers divide HCs regeneration process into three characteristic modules by the transcriptome analyses. When HC facing the damage, progenitor maintenance/placodal genes including Notch and Fgf signaling are initially downregulated, accompanied by activation of injury/inflammatory response and glucocorticoid signaling. Subsequently, MAPK cascade and NOD-like receptor signaling began to enrichment. In the following HC regeneration stage, ribosome biogenesis genes, epigenetic regulators (e.g., *seta*, *arid3b*, and *hdac3*) and HC lineage genes such as *atoh1a* are highly expressed in young HCs (Baek et al., 2022). Using the H3K9me2 inhibitor BIX01294 will reduces zebrafish HCs regeneration ability and downregulates Wnt and Fgf signaling pathways that crucial for HCs regeneration (Tang et al., 2016).

Although sensory HCs cannot regenerate spontaneously after death in the mammalian inner ear, researchers have observed the presence of stem cells in the sensory epithelium of the adult mouse utricle. These cells possess high proliferative potential and self-renewal capacity and exhibit the characteristics of adult stem cells. A portion of the sphere-derived stem cells showed characteristics of the HC phenotype, such as expressing HC markers, F-actin-rich specializations and the absence of SC markers. This raises hope for the generation of HCs by modifying the signal transduction pathways to control adult inner-ear stem cell proliferative behavior (Li et al., 2003). In rapidly proliferating tissues, the *Wnt* target gene *Lgr5* has been demonstrated to identify endogenous stem cells. *In vitro* cultured cochlear sensory epithelial cells showing that Lgr5^+^ SCs behave similarly to sensory progenitor cells. Overactive Wnt signaling could allow Lgr5^+^ cells to break cycle arrest and proliferate temporarily *in vivo*. *In vitro*, Lgr5^+^ cells are induced to proliferate by active Wnt signaling. Wnt agonists increased Myo7a^+^ cell numbers in Lgr5^+^ cell-formed colonies, and Wnt inhibitors diminished the proliferative capacity of Lgr5^+^ cells. This result shows that Wnt signaling may serve as a potential target in HC regeneration since Lgr5-marked SCs act as sensory precursor cells both *in vitro* and *in vivo* (Chai et al., 2012). The Notch signaling pathway is crucial for HC differentiation during inner ear development, and its activities include two different modalities: lateral induction and lateral inhibition. Activated Notch signaling could prevent SC to HC conversion during HC development. In the neonatal cochlea, gentamicin treatment could induce HC loss and could be considerably restored by *Notch* inhibition, mainly due to Notch inhibition activating SC transdifferentiation into HCs (Bramhall et al., 2014). In the noise-induced hearing loss model, Notch signaling inhibition induced *Atoh1* overexpression, which promoted SC transdifferentiation into HCs after noise trauma. After HC regeneration in the adult cochlea by Notch signaling inhibition, the hearing loss caused by noise exposure will recover (Mizutari et al., 2013). After downregulating ephrin-B2 signaling during embryonic stages, SCs could move into HC layers and subsequently switch their identity from SC to HC fate. It has been suggested that ephrin-B2 signaling may exhibit local inhibition from SC to HC transdifferentiation (Defourny et al., 2015).

With the gradual progress in HC regeneration research, researchers have found that coordinated regulation with multiple pathways could synergistically promote HC regeneration. Hippo signaling inhibition could promote SC proliferation and differentiation into HCs in the neonatal cochlea after neomycin damage. As a key Hippo pathway downstream mediator, YAP nuclear accumulation can promote direct SC transdifferentiation and induce supernumerary HC generation after ototoxicity damage in neonatal mice. Furthermore, turning off Hippo with *Notch* inhibition could significantly increase HC differentiation compared with single-pathway regulation. This synergistic effect is especially shown in promoting the direct differentiation of SCs into HCs (Lu et al., 2022). In the neonatal cochlea, constitutively active β-catenin and ectopically expressing Atoh1 could increase HCs by synergistic proliferation and differentiation of Lgr5^+^ cells (Kuo et al., 2015). As two classical pathways that determine the fate of inner ear cells, Wnt and Notch signaling play important roles in regulating cell proliferation and differentiation. The combination of Wnt activation and Notch1 deletion could induce massive Sox2^+^ SC proliferation from the apex to the base, which offers an effective strategy for activating HC generation at the basal turn (Ni et al., 2016). These results demonstrated that Notch inhibition caused Sox2^+^ SCs to upregulate β*-catenin* and activate *Wnt* downstream target genes. Notch inhibition could initiate SC proliferation and HC mitotic regeneration and transdifferentiation *in vivo* and *in vitro*. The results illustrate that Notch inhibition stimulates the canonical Wnt pathway in cochlear sensory epithelium progenitor cells and proceeds in mitotic production of HCs (Li et al., 2015).

## The epigenetic mechanisms of HC regeneration

Studies have shown that iPSC reprogramming efficiency can be improved through the regulation of DNA methylation and epigenomic reprogramming (Kim et al., 2010; Lister et al., 2011). Unlike mammalian HCs, HCs in avians can be regenerated after injury. SCs from avian cochlear epithelium will proliferate and direct cellular conversion to HCs when damage occurs (Layman and Zuo, 2015). Histone deacetylases (HDACs), as histone acetylation regulators, play an important role in the regulation of regenerative proliferation in the chick utricle. Utricle cultures treated with HDAC inhibitors will result in decreased SC proliferation. However, HDAC inhibitor treatment does not affect replacement HC differentiation (Slattery et al., 2009). In zebrafish lateral line development, downregulation of histone demethylase lysine-specific demethylase 1 (LSD1) inhibits cell proliferation and reduces HC formation. Furthermore, after neomycin-induced HC loss, LSD1 inhibition significantly inactivates the Wnt/β-catenin and Fgf signaling pathways, which are responsible for HC regeneration (He et al., 2016). Knockdown of kdm6bb, a H3K27 demethylase, will also reduce Fgf signaling pathway-related gene expression and increase the *axin2* and *lef1* expression levels of Wnt/β-catenin signaling during zebrafish neuromast development (Tang et al., 2021). The data show that histone deacetylation plays a critical role in the regulation of HC regenerative proliferation. However, the role of histone modification after mammalian HC injury is still unclear. Identifying the epigenetic regulation mechanisms after mammalian cochlear HC injury would provide important information to complete mammalian HC regeneration (Layman and Zuo, 2015). The low number of induced *Lgr5*^+^ cells in the damaged cochlear sensory epithelium is one of the aspects that restricts HC regeneration from SC transdifferentiation. Researchers have demonstrated that *Lgr5*^+^ cells from the inner ear could be significantly enlarged using a combination of the HDAC inhibitor valproic acid to activate Notch signaling and a GSK3 inhibitor to stimulate the Wnt signaling pathway. In turn, these *Lgr5*^+^ cell colonies could be effectively separated into nearly pure populations of HCs (McLean et al., 2017).

Combinatorial regulation of three transcription factors, *Atoh1*, *Gfi1* and *Pou4f3*, may effectively reprogram non-sensory cells to take on an HC fate in the neonatal cochlea. However, the expression of these transcription factors is decreased by reprogramming with increasing age. Multiomic analysis showing that ATOH1 binding sites and the *Pou4f3* locus become less epigenetically accessible after the postnatal period. This finding provides evidence that epigenetic modulation affects the HC regeneration program after birth by regulating transcription factor accessibility (Iyer et al., 2022). *Atoh1* expression is associated with SC differentiation and is downregulated during HC maturation. The regulation of *Atoh1* expression was dependent on histone acetylation, and H3K9ac levels significantly increased at the *Atoh1* locus between E14.5 progenitors and E17.5 HCs. When HCs gradually mature, *Atoh1* decreases correlate with decreased levels of H3K9ac (Stojanova et al., 2015). H3K4me1 was confirmed as a marker of active enhancers in SCs that could positively regulate enhancer activity by chromatin remodeling, recruit the Cohesin complex and prevent permanent silencing of DNA methylation of enhancers. Chromatin remodeling and Cohesin recruitment will make primed enhancer genes more accessible and allow interactions between enhancers and target gene promoters for actively regulated gene transcription. The loss of H3K4me1 during cochlear maturation leads to the loss of SC regenerative potential. With the maturation of SCs, DNA methylation and inhibitory histone marks, H3K27me3 and H3K9me3, increase at HC gene loci. Furthermore, they used DAPT, a Notch inhibitor, to determine the epigenetic landscape as SCs transdifferentiate. Open chromatin and H3K27ac marks were considerably higher in transdifferentiating SCs, which indicates that primed enhancers facilitate SC reprogramming (Tao et al., 2021). As an active and dynamic histone methylation marker, H3K4me3 modification sites were significantly changed in Lgr5^+^ progenitor cells after neomycin treatment. H3K9me3 modification affects the expression of genes associated with HC proliferation and regeneration. Enrichment analysis showed that differentially expressed H3K4me3 binding sequences can dramatically enrich three transcription factor (*Zeb1*, *Fev*, and *Prdm5*) motifs. These transcription factors perform regulatory activities for the proliferation and regeneration of HCs by affecting the regulation of their target genes (Ma et al., 2022).

By regulating DNA epigenetics, progenitor cells in the inner ear will change their proliferation and transdifferentiated abilities. Using 5-azacytidine (5-aza), a DNA methyltransferase (DNMT) inhibitor, to treat mouse utricle sensory epithelia-derived progenitor cell lines (MUCs) will downregulate *Dnmt* gene and protein expression, which can cause DNA demethylation. The consequent changes after 5-aza administration will induce sensory HC markers *Atoh1*, *Myo6* and *Pou4f3* expression in MUCs. The promoter regions of the *Cdh1, Atoh1*, and *Pou4f3* genes are demethylated after 5-aza treatment and activate HC gene and protein expression in MUCs, finally inducing MUC differentiation into hair cell-like cells. In addition, treated MUCs will exhibit some properties of mechanotransduction channels of HCs (Zhou and Hu, 2016). Furthermore, 5-aza was used when young adult mice were exposed to kanamycin plus furosemide treatment. Compared with saline treatment, 5-aza inner ear injection induced a significant number of regenerated HCs after ototoxic drug treatment. These newly generated HCs survived for at least 6 weeks after 5-aza therapy. Quantitative PCR results indicate that 5-aza-induced HC regeneration may be related to *Dnmt1* downregulation. Notch and Wnt signaling, two key signaling pathways related to HC regeneration, were also downregulated after 5-aza treatment (Deng et al., 2019).

## Conclusion

**FIGURE 1:**
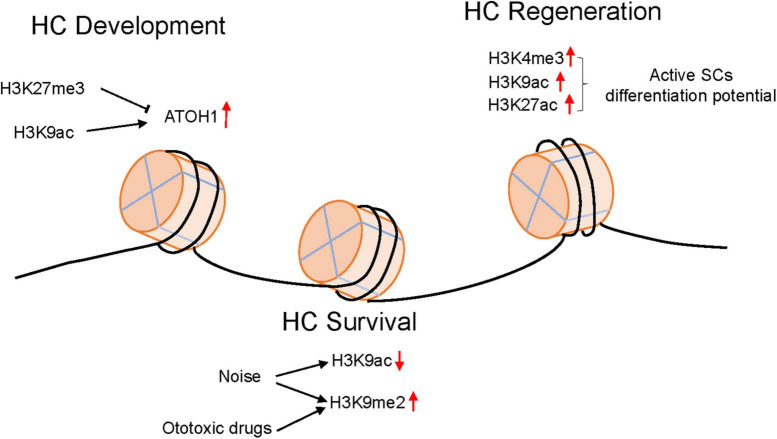
The epigenetic modification changes in HCs in different environments.

Epigenetic modification significantly influences gene expression and translation. Epigenetic modification was first thought to be an irreversible form of chromatin modification. Many reversible types of epigenetic modifications have been discovered recently, providing a new perspective for understanding the mechanism of ototoxic drug damage, noise-induced hearing loss and presbycusis (Figure 1). Similarly, through epigenetic regulation, we provide a new opportunity to prevent hearing loss caused by numerous factors. In cancer therapy, some of epidrugs that identification and targeting of epigenetic modification enzymes have been approved by FDA for tumor treatment. The emergence of epidrugs for epigenetic alteration in the process of bone regeneration and repair also brings hope for the treatment of metabolic bone disorders. However, epidrugs are not available to hearing loss treatment. Further studies are needed to explore the specific epigenetic marker changes in HCs when suffering from ototoxic factors stimulation. Furthermore, how the epigenetic modifications affect the signaling changes in HCs also needs to be clarified. The HC regeneration signaling pathways were also greatly regulated by epigenetic regulation. It is believed that further studies on the epigenetic regulation network will provide more measures for hearing loss prevention.

## Author contributions

YX and DL wrote the manuscript. DL reviewed and edited the manuscript. Both authors approved the manuscript for publication.
